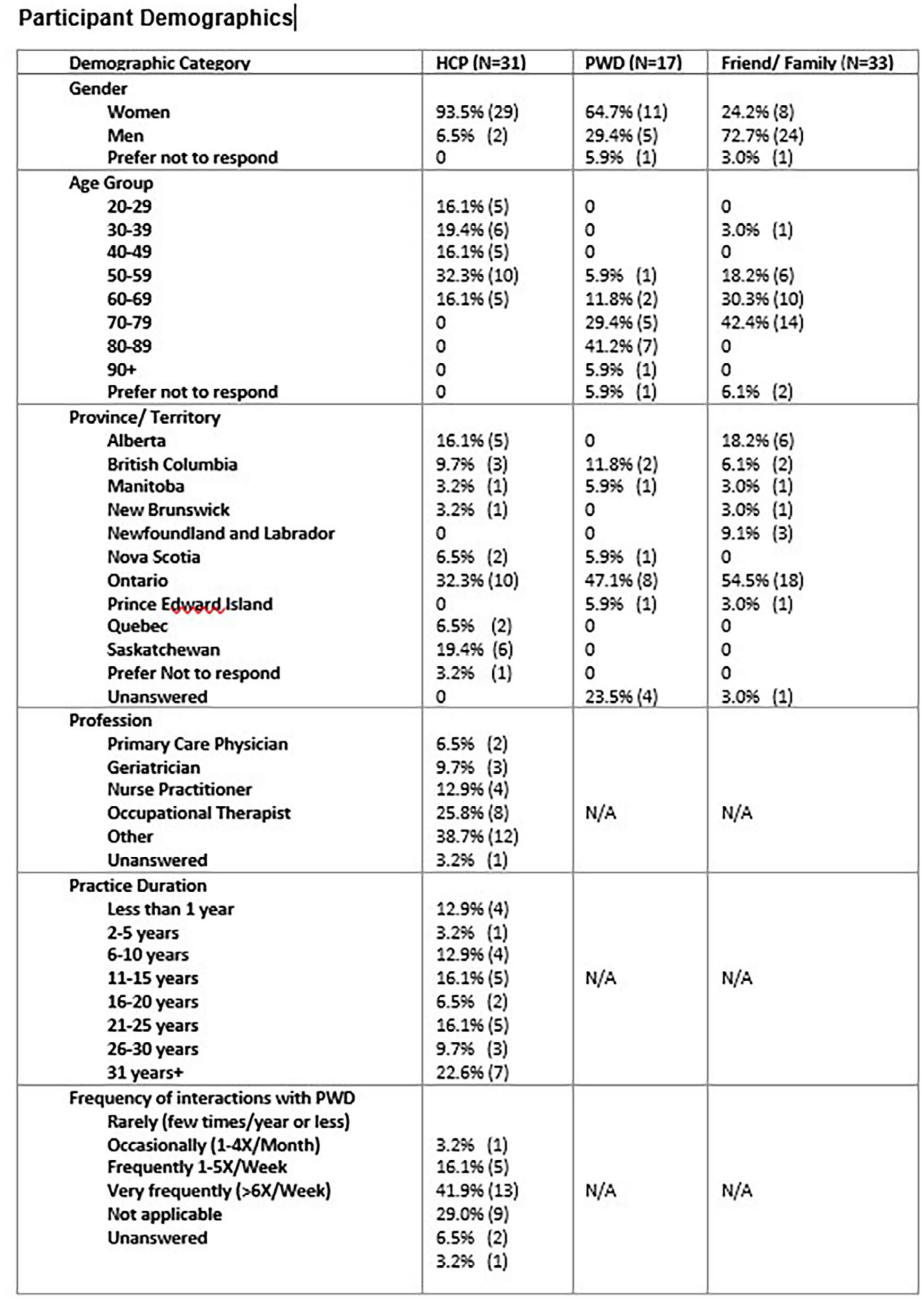# Exploring users' perspectives of the Driving and Dementia Roadmap

**DOI:** 10.1002/alz.091035

**Published:** 2025-01-09

**Authors:** Gary Naglie, Elaine Stasiulis, Mark J. Rapoport, CCNA Driving Cessation Team Co‐Investigators and CCNA Study Group

**Affiliations:** ^1^ Baycrest Health Sciences, Toronto, ON Canada; ^2^ University of Toronto, Toronto, ON Canada; ^3^ Rotman Research Institute, Toronto, ON Canada; ^4^ Sunnybrook Health Sciences Centre, Toronto, ON Canada

## Abstract

**Background:**

Since October, 2022 the Driving and Dementia Roadmap (DDR) (www.drivinganddementia.ca) – an online resource to support people with dementia (PWD), family/friend carers (FCs) and healthcare providers (HCPs) as they navigate the challenges of driving cessation – has been accessed by over 34,000 users. To understand the DDR’s impact we are conducting on‐going surveys to explore users’ perspectives of the DDR.

**Method:**

As users exit the DDR, they are invited, via pop‐up message, to participate in an online survey about their experiences with the DDR, including their perceived new knowledge and confidence gained due to using the DDR and their satisfaction with it. Descriptive statistics were conducted via the REDCap platform.

**Result:**

To date, 81 DDR users have participated in the survey (17 PWD, 33 FCs and 31 HCPs). Topics rated the highest as “new knowledge gained” included: for PWD, “recognizing unsafe driving” (17.6%) and “getting around without driving” (17.6%); for FCs, “recognizing unsafe driving” (59.4%) and “learning about licensing and reporting” (59.4%); and for HCPs, “providing support after driving cessation” (61.3%) and “having discussions and managing emotions” (45.2%). With regards to increases in confidence reported as “somewhat” to “much more confident”, the highest rated topics included: for PWD, “making the decision to stop driving” (47.1%) and “recognizing unsafe driving” (41.2%); for FCs, “making the decision to stop driving” (71.9%) and “initiating conversations about driving cessation” (68.8%); and for HCPs, “managing the emotional impact” (71.1%) and “having conversations about driving cessation” (67.7%). Satisfaction levels by participants across all DDR characteristics were rated as “satisfied” to “very satisfied” by at least 47% of PWD, 87% of FCs and 80% by HCPs, with “trustworthiness of the information” rated the highest by all three groups (PWD: 75.1%; FCs: 93.8%; HCPs: 87.1%).

**Conclusion:**

Early results indicate that a majority of users were satisfied with the DDR and using the DDR led to gains in new knowledge and increased confidence in managing aspects of driving cessation for all 3 groups, but least so for PWD. The next step will involve in‐depth interviews with participants to better understand the user experience and the relatively lower ratings among PWD.